# Implications of Oncology Trial Design and Uncertainties in Efficacy-Safety Data on Health Technology Assessments

**DOI:** 10.3390/curroncol29080455

**Published:** 2022-08-16

**Authors:** Dario Trapani, Kiu Tay-Teo, Megan E. Tesch, Felipe Roitberg, Manju Sengar, Sara C. Altuna, Michael J. Hassett, Armando A. Genazzani, Aaron S. Kesselheim, Giuseppe Curigliano

**Affiliations:** 1 European Institute of Oncology, IRCCS, 20141 Milan, Italy; 2 Department of Medical Oncology, Dana-Farber Cancer Institute, Boston, MA 02215, USA; 3 Department of Pharmaceutical Sciences, Università Del Piemonte Orientale, 28100 Novara, Italy; 4 Department of Health Products Policy and Standards, World Health Organization, 1211 Geneva, Switzerland; 5 Medical Oncology Service, Hospital Sírio-Libanês, São Paulo 01308-050, Brazil; 6 Tata Memorial Centre, Homi Bhabha National Institute, Mumbai 400094, India; 7 Oncomédica C. A., Caracas 1040, Venezuela; 8 Program on Regulation, Therapeutics, and Law (PORTAL), Division of Pharmacoepidemiology and Pharmacoeconomics, Department of Medicine, Brigham and Women’s Hospital and Harvard Medical School, Boston, MA 1620, USA; 9 Department of Oncology and Hemato-Oncology, University of Milan, 20122 Milan, Italy

**Keywords:** cancer medicines, health technology assessment, value, dis-investments, MCBS, health economics, accelerated approval

## Abstract

Background: Advances in cancer medicines have resulted in tangible health impacts, but the magnitude of benefits of approved cancer medicines could vary greatly. Health Technology Assessment (HTA) is a multidisciplinary process used to inform resource allocation through a systematic value assessment of health technology. This paper reviews the challenges in conducting HTA for cancer medicines arising from oncology trial designs and uncertainties of safety-efficacy data. Methods: Multiple databases (PubMed, Scopus and Google Scholar) and grey literature (public health agencies and governmental reports) were searched to inform this policy narrative review. Results: A lack of robust efficacy-safety data from clinical trials and other relevant sources of evidence has made HTA for cancer medicines challenging. The approval of cancer medicines through expedited pathways has increased in recent years, in which surrogate endpoints or biomarkers for patient selection have been widely used. Using these surrogate endpoints has created uncertainties in translating surrogate measures into patient-centric clinically (survival and quality of life) and economically (cost-effectiveness and budget impact) meaningful outcomes, with potential effects on diverting scarce health resources to low-value or detrimental interventions. Potential solutions include policy harmonization between regulatory and HTA authorities, commitment to generating robust post-marketing efficacy-safety data, managing uncertainties through risk-sharing agreements, and using value frameworks. Conclusion: A lack of robust efficacy-safety data is a central problem for conducting HTA of cancer medicines, potentially resulting in misinformed resource allocation.

## 1. Introduction

The past decades have seen remarkable progress in cancer care because of advances in prevention, treatment, and palliative care. The advent of some new classes of cancer medicines has redefined treatment paradigm with transformative impacts on patient health outcomes [1]. However, a significant proportion of cancer medicines approved over the past decades demonstrated varying levels of benefit, with many medicines showing no to low additional benefits to patients compared to the existing standard of care, despite having received regulatory approval [2]. Some cancer medicines can also cause severe adverse events for patients, further complicating the benefit-risk balance of their use in clinical practice [3]. In parallel, cancer medicines often incur high costs, challenging the financial sustainability of health systems globally, including in high-income countries [4,5]. For individual patients, high-cost cancer medicines expose them to the risk of catastrophic health expenditure, especially patients living in health systems with poor social protection.

In the context of competing health priorities, health system designs and the ambition of providing universal health coverage, there have been ongoing policy debates about how best to ensure affordable access to effective cancer medicines and improve health system efficiency while incentivizing enterprise and innovation [6,7]. To this end, many countries have turned to Health Technology Assessment (HTA) to inform system-wide decision-making better [8]. Specifically, HTA has been used to tailor the allocation of resources toward interventions of higher value so that health and societal outcomes can be maximized according to the local population’s needs at a cost representing value for money [9], as shown in Table 1.

Conducting HTA is inherently data-intensive, and one of its key prerequisites is robust data collected from pivotal clinical trials. When data is insufficient or inadequate, assumptions are needed in HTA for estimating an intervention’s benefits, costs, cost-effectiveness and budget impacts. Depending on the set of assumptions, such assumptions could introduce substantial uncertainties, rendering the findings of HTA unreliable and potentially directing resources towards lower-value and non-priority interventions. These low-value interventions could yield worse population health outcomes and cause inefficient resource allocation, defeating the very purpose of undertaking HTA [10,11,12,13,14,15].

In recent years, the quest to facilitate early access to new cancer medicines through fast-track regulatory pathways and other regulatory flexibilities has attracted considerable debates about the robustness of clinical trial designs and data [16]. These issues have further implications for HTA and by extension, health outcomes and resource allocation. Thus, it is important to clarify how clinical trial designs and data limitations would affect HTA.

To this end, this paper presents a literature review to describe selected challenges of clinical trial designs and data in oncology as they are applied to HTA of cancer medicines. It first briefly presents what HTA encompasses for health technologies in cancer. It then addresses specific challenges, including translating surrogate measures into clinically and economically meaningful outcomes, using single-arm and basket clinical trials, and lacking data to inform HTA. This paper concludes with suggestions for consideration when conducting HTA of cancer medicines.

### Literature Review

**Figure 1:**
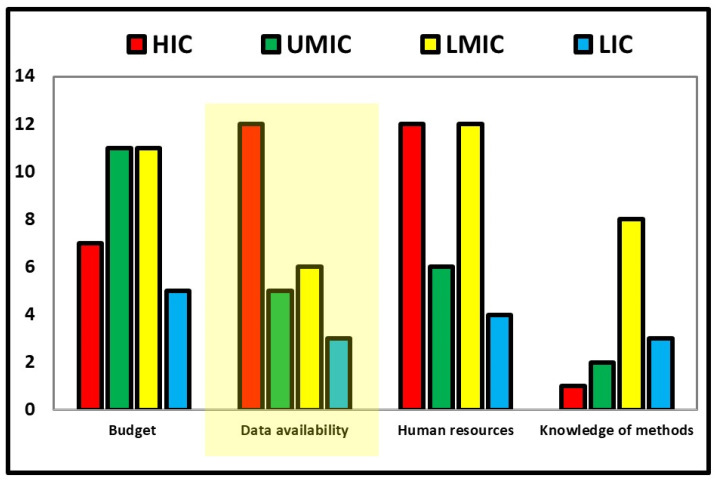
Principal barriers of conducting Health Technology Assessment in countries. HIC: high-income countries; UMIC: upper-middle-income countries; LMIC, lower-middle-income countries; LIC: low-income countries. Source: WHO Health Technology Assessment Survey 2020–2021. Source: [17].

This is a narrative policy review. A search of peer-reviewed literature from PubMed, Scopus and Google Scholar for “HTA” and “cancer medicines” was conducted. Online resources of grey literature from international public health agencies, including United Nations agencies, governmental HTA bodies and Ministries of Health were also consulted. The literature was initially reviewed to identify potential gaps and challenges in conducting HTA. Initially, we focused on non-structural barriers in implementing HTA (i.e., structural barriers are those related to a lack of skilled human resources or budget and others requiring structural investments) by expanding on the determinants of uncertainties when conducting HTA. Preparatory research for this paper (DT) identified the lack of quality data on efficacy-safety as the major non-structural barrier to conducting HTA at the global level (Figure 1). Accordingly, this is the primary focus of this paper.

## 2. What Does HTA Mean When Applied to Cancer Medicines?

### 2.1. Defining HTA

**Figure 2:**
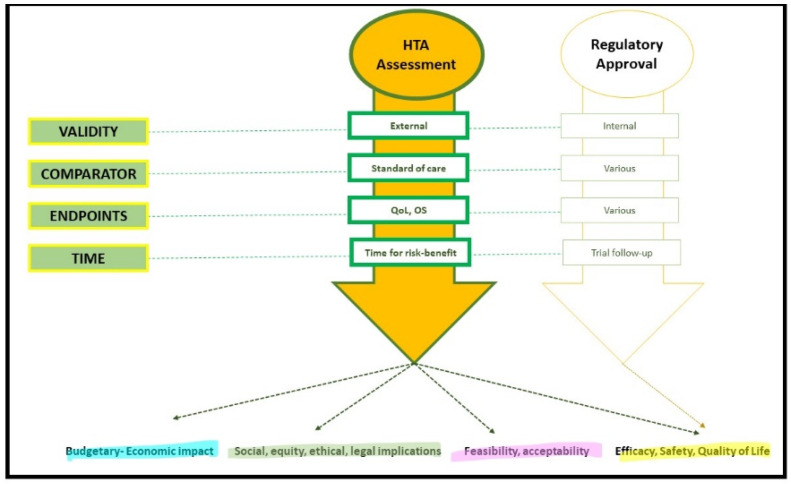
Structural elements and outputs of the Health Technology Assessment process versus drug approval regulation. QoL, quality of life. OS, overall survival. Note: the comparator for HTA can be a local standard of care and not mirror an international standard of care, as in the purpose of HTA. Regulatory agencies can account to some extent for the real world-evidence, with interest in the external validity of the clinical trial findings. Time for risk-benefit is intended as the interval of time deemed appropriate to consider the information on the outcome mature enough to draw conclusions; for example, in the case of overall survival in oncology clinical trials, this interval is commonly 3 to 10 years, according to the specific disease type and setting.

HTA has been defined as “a multidisciplinary process that uses explicit methods to determine the value of health technology at different points in its lifecycle”, with the purpose of “informing decision-making in order to promote an equitable, efficient, and high-quality health system” [6,18]. Figure 2. In cancer, health technology encompasses a broad range of health interventions, including medicines, ranging from small molecules and complex biologicals to cell- and gene-based therapies. The use of many precision oncology medicines requires a complex set of clinical services, including companion diagnostics. These non-pharmaceutical products would need to be accounted for when assessing the overall value of new cancer medicine, thereby increasing the complexity of HTA.

### 2.2. Conceptualizing Value in HTA 

In HTA, the notion of value is broadly conceived. Value encompasses clinical effectiveness; safety, costs, and economic implications; ethical, social, cultural and legal issues; and organizational and environmental aspects, as well as wider implications for the patient, relatives, caregivers, and the population [14,19]. In practice, value in HTA is frequently (mis)interpreted as the cost-effectiveness of a technology [20]. It is important to recognize the broad dimensions of value beyond clinical and economic values and that some important dimensions cannot be readily enumerated quantitatively [21]. Some argue that HTA and regulators address divergent questions to deliver patient-centric and population health benefits, therefore conceptualizing value based on divergent assumptions. Notably, HTA is devoted to estimates of population health impact, but overall framed in a person-centered approach, thus based on the trade-off to offer both broader public health and individual patient benefits. This is particularly pertinent for cancer medicines because resources may be allocated to meet other social goals (e.g., social value for end-of-life care, equitable access) that may not fully align with the goal of maximizing health outcomes or economic efficiencies–that is commonly critical to deliver equitable care and health for all, but should not be a restriction in the uptake of high-value interventions. Accordingly, the dialogue generated by HTA should not be reduced to a question of costs or cost-effectiveness alone, but framed across the comprehensiveness of HTA, to formulate, and understand the ultimate outcomes of HTA, value-driven recommendations. At the end, decision-makers are called to reconcile any misalignments in quantifiable (e.g., cost-effectiveness) and non-quantifiable value (e.g., impact on equitability), based on a comprehensive approach. 

### 2.3. When HTA Can Be Applied 

HTA can be applied at different points in the lifecycle of health technology, from the pre-market phase (e.g., when specifying target product profiles), during assessment for market approval, and post-market to eventual disinvestments. Reassessment of value is particularly relevant for cancer medicines because of the rapidly changing therapeutic landscape and standard of care and the considerable budgetary impacts of cancer medicines. It is also essential to re-assess the HTA of marketed medicines as country regulators and payer organizations have become more willing to grant early access to new medicines based on preliminary evidence of positive benefit-risk profiles on the condition that additional evidence will be generated [22].

### 2.4. Resource Requirements of HTA 

HTA requires considerable human and financial resources for managing technical, administrative and governance issues during implementation. HTA also requires robust information technology infrastructure and reliable data (cancer registries and administrative databases). In recent years, countries have developed a considerable capacity to support HTA implementation, particularly following the World Health Organization’s resolution WHA67.23 on health intervention and technology assessment in support of universal health coverage [23]. It must be recognized that the ability to undertake robust HTAs in resource-constrained settings—where it might also be most helpful in informing efficient allocation of scarce resources—paradoxically remains significantly underdeveloped [24].

## 3. Challenges Relating to Clinical Trial Data

### 3.1. Translating Surrogate Measures into Clinically and Economically Meaningful Outcomes in HTA

#### 3.1.1. About Surrogate Measures

Surrogate measures are laboratory values or radiologic measurements used in place of direct clinical outcomes, such as how long a patient survives, feels or functions (quality of life (QoL), patient-reported outcomes (PROs)) [25,26,27]. The use of surrogate measures–such as progression-free survival (PFS), disease-free survival (DFS), objective response rate (ORR), minimal residual disease (MRD) and many others–in clinical trials of cancer medicines has been widespread [28,29]. 

Surrogate measures are used in clinical trials of investigational cancer medicines to facilitate earlier regulatory approval because they can be measured sooner and with smaller sample size requirements and lower investments for drug development than “direct” clinical endpoints, such as overall survival (OS) and QoL. Moreover, they can be preferred in the context of rare tumors and rare genetic alterations in common tumors and for diseases with long post-trial survival, when several post-progression lines of treatment could jeopardize or dilute the ultimate OS benefit. In a key example, for a third of the medicines investigated in a study using the “PACE Continuous Innovation Indicators” platform, the first regulatory approval based on surrogate metrics was at least 4 years earlier than when OS evidence was published [30].

Regulatory agencies worldwide have accepted surrogate endpoints as the primary evidentiary basis for granting marketing authorization [31]. For example, the Food and Drug Administration (FDA) in the United States (US) granted two-thirds of the marketing authorizations of cancer medicines approved between 2009 and 2014 based on surrogate measures, while all cancer medicines approved via the US FDA’s accelerated approval regulatory pathway are by definition based on surrogate endpoints [31,32,33]. 

#### 3.1.2. Improved Surrogate Endpoints Do Not Always Mean Better Outcomes

Surrogate measures are problematic when they have not been fully validated as predictive of clinically-meaningful and patient-centric outcomes [34]. Specifically, improvements in surrogate endpoints will not necessarily translate into better OS and QoL—the gold standard endpoints, according to several regulatory and reimbursement agencies [35]. In an analysis of 47 US FDA approvals for cancer medicines made between April 2014 and February 2016, only 19% of therapies approved showed improvement in OS at the time of approval [36]. Another analysis showed that of the 36 medicines approved by the US FDA based on surrogate endpoints, at a median follow-up of 4.4 years, 18 medicines failed to improve OS as primary or secondary outcomes in randomized studies, and the effects of 13 medicines continued to be unknown [30,37,38]. From 2011–2018, the European Medicines Agency (EMA) approved 51 products via accelerated or conditional approvals [39]. Of them, 61% of the decisions were supported by endpoints of “reasonable” surrogacy value, and 94% had “biological plausibility” to predict clinical outcomes, with no OS mature data yet available. 

#### 3.1.3. Introduction of Uncertainties When Translating Surrogate Measures in HTA

When ascertaining the benefits of cancer medicines through HTA, improvements in surrogate measures would need to be translated into OS or QoL estimates, extrapolated over time and inform key metrics for HTA, including cost-effectiveness. Such transformation and extrapolation can add substantial uncertainty to the assessment, as their robustness is subject to the assumptions used and the selected methodologies [40,41,42,43,44]. For example, a methodology review assessing the validity of PFS as a surrogate endpoint for predicting OS in cancer clinical trials found wide heterogeneity in methods and reporting [29]. In addition, data on QoL and PROs are often not collected in or not reported by pivotal clinical trials [45,46,47,48,49,50]. 

HTA agencies also lack clear and consistent guidance on evaluating surrogate measures when considering coverage, pricing, and reimbursement decisions. In a recent review, half of the guidance from HTA agencies stated the acceptability of surrogate endpoints for HTA purposes. Still, fewer than 20% of the guidelines reflected a clear difference between surrogate and direct endpoints [51,52].

#### 3.1.4. Uncertainties Could Lead to Adoption of Suboptimal Interventions and Misallocation of Resources

The uncertainties associated with surrogate measures could lead to the uptake of low-value interventions at costs that do not align with their true value. A recent study evaluated the budget implications of selected medicines approved through accelerated pathways, which subsequently failed to show a benefit on patient outcomes [53]. The investigators surveyed six cancer indications for four medicines and estimated that these indication-medicine combinations incurred US$224 million in expenditure between 2017 and 2019 to US Medicare [54]. In 2016, the US FDA granted atezolizumab accelerated approval for use in patients with advanced urothelial carcinoma who had previously received platinum-based chemotherapy based on surrogate endpoints [55]. The approval was based on early evidence of ORR benefits gathered in a single-arm trial. A subsequent Phase III clinical trial failed to demonstrate improvements in OS, resulting in the withdrawal of the indication [56]. A similar situation occurred with another immune checkpoint inhibitor for advanced urothelial cancer, durvalumab, which was withdrawn in 2021 [56]. In the context of rare diseases, uncertainty might be more readily accepted when considering regulatory decisions and be granted accelerated approvals to address high unmet clinical needs [57,58,59]. For example, the PDGFRα antagonist olaratumab was granted accelerated approval for treating patients with advanced soft tissue sarcoma based on data from the phase II JGDG study [60]. The JGDG trial reported an absolute improvement in the median PFS of 2.5 months with the addition of olaratumab, along with a substantially longer OS gain of 11.8 months, which was unprecedented in this setting. The medicine was subsequently withdrawn in 2019 because the phase 3 clinical trial ANNOUNCE failed to demonstrate any improvement in OS [61]. In reflection, the initial approval was based on highly uncertain data in a disease setting with a poor prognosis. Despite the lack of health impacts, the decision generated sales income of more than $200 million USD in 2017 and 2018 for the company, which corresponded to a sizeable budgetary impact for the payers [62]. In summary, based solely on surrogate measures for regulatory approval, these medicines were subsequently covered and reimbursed in the US Medicare program for years, yielding minimal benefit if not causing harm, with unclear QoL gains, and at a significant expense and opportunity costs to the health system. In all these cases, a lack of robust safety-efficacy data misled the reimbursement and coverage decisions, leading to the wastage of public and private funds [57]. In settings where scarce resources are allocated for healthcare, specifically for cancer control, such uncertainties can significantly impact the development and sustainability of cancer programs [63].

### 3.2. Generalizability of Clinical Trial Findings to the Specific Contexts of Use

A major aim of HTA is to evaluate the benefits and costs of new medicine in a context-specific manner, to address local health needs. HTA aims to assess the generalizability of clinical trial results of medicines with consideration to various factors influencing the circumstances of use, such as target population, existing standard of care, and availability of certain clinical services [64,65]. Any mismatches between the trial population and the overall patient population present a challenge for undertaking HTA [66]. For example, a systematic evaluation of the pivotal clinical trials leading to US FDA approval of cancer medicines showed major gender and racial under-representation (e.g., African-Americans) compared to data from cancer epidemiology [67]. In addition, despite representing over 40% of the overall cancer population, patients aged ≥70 years are commonly underrepresented in clinical trials. Those enrolled in clinical trials typically have fewer comorbidities and functional impairments than in real life [67,68]. A further transferability issue derives from the geographical distribution of enrolled patients and poorer performance status than in pivotal clinical trials [67].

Outcomes in patients who experience different standards of care are also a challenge for HTA [69,70,71]. For example, under-representation of clinical trials in low-income and middle-income countries, where resources are most constrained, and standards of care are often different, could also result in misleading value assessment if clinical trial evidence or HTA findings from high-income countries were to be directly translated into the decision-making, without due consideration for key differences in patient populations, health systems and circumstances of use [72]. While some countries may have neither the data nor the infrastructure to support the translation of evidence into the specific local context of use, such considerations must be explicitly accounted for when making decisions based on HTA [73,74]. 

## 4. Challenges Arising from Clinical Trial Designs

### 4.1. Single-Arm Trials 

In recent years, evidence from single-arm trials (SATs) has frequently formed the basis of regulatory approval. In a single-arm trial, statistical approaches have been used for comparing efficacy endpoints indirectly against a historical control or descriptive studies with no comparisons [75,76,77]. Over the last two decades, the US FDA has granted approvals for 153 new indications based on SATs, of which two-thirds went through an expedited pathway [75]. SATs have been used for many types of cancer medicines; however, some therapeutics, like cell- and gene-based therapies, including CAR-T therapy, have been exclusively tested in SATs. 

The main justification for accepting SATs as the only evidence supporting a positive regulatory decision is the existence of a high clinical unmet need if no other alternatives exist for treating a severe illness. The medicine has shown a likelihood of conferring beneficial therapeutic effects. Some have argued that historical control arms could be developed from previous trials, evidence from routine clinical care or registries, or health claims or electronic health records to complement missing data and fill relevant knowledge gaps in SATs [76,78]. However, evidence generation based on such data may not always be feasible, and even when able to be gathered, these data usually present strong methodological bias (for example, selection bias) that cannot mitigate the high level of uncertainty [64,76,79,80,81]. 

HTAs based on SATs do not always include external comparators–an important limitation considering the comparative characteristics of HTA. A report analyzing SAT-based HTAs from 21 countries showed that 40% of the HTAs submitted for reimbursement approval did not include any external comparator. In contrast, the remaining 60% of the studies included descriptive external comparators, with no formal statistical assessment of the comparative effectiveness versus the standard of care treatments [64]. Another study examined 22 medicines evaluated by the United Kingdom’s National Institute for Health and Care Excellence between 2000 and 2016 that used non-randomized controlled trial data to estimate comparative clinical effectiveness. This study found that 23% of the HTAs included adjustments for co-variates through regression analysis when estimating the comparative benefits. Other comparisons were either unadjusted for co-variates or not methodologically robust [82].

Using SATs to support regulatory decisions can also hamper the ability to conduct confirmatory randomized clinical trials subsequently. This can happen because clinicians would be more reluctant to enroll patients to participate in the confirmatory trials when the medicine under investigation is already available for clinical use to their patients, perceiving the control arm as not their contemporary standard of care.

In summary, SAT study design not only introduces uncertainties on efficacy-safety but also further affects the robustness of HTA because of a lack of a formal comparison arm to assess the incremental costs and benefits. SAT also limits or delays the generation of robust data in future confirmatory trials. Therefore, HTA bodies must deal with highly uncertain and mostly descriptive data to inform recommendations, with a higher likelihood of making misinformed decisions. These challenges are even more pronounced in the context of rare tumors, including childhood cancers, of the issues in completing enrolment in large clinical trials.

### 4.2. Basket Clinical Trials and Histology-Agnostic Indications 

Basket clinical trials have emerged in recent years to study the effects of medicine targeting certain molecular characteristics in a group, or “basket”, of patients with different tumors but common actionable genetic alterations. New trial designs have tested cancer medicines based on actionable mutations, regardless of the tumor histology, on the assumption of histology-tumor agnosticism. Agnostic indications are approved across cancer types insofar as the tumor harbors a specific molecular characteristic. For example, the US FDA approved tissue-agnostic indication for pembrolizumab based on tumor mutational burden (TMB) as the predictive marker [83].

However, it has been reported that tumor responsiveness can vary by tumor type across several histologies. In the case of pembrolizumab, the pivotal KEYNOTE-158 clinical trial showed that some tumors with high TMB were refractory to pembrolizumab, while others could derive benefits despite low TMB [84]. These differences in the magnitude of clinical benefits, or the lack thereof, and the lack of comparative data on the tumor-specific outcomes, yield important complexities when undertaking HTA to inform coverage, pricing and reimbursement decisions for histology-agnostic indications considering that the benefits expected are variable by tumor histology. The benefits are unpredictable in tumor tissues with no data from the clinical trial [85,86]. Therefore, histology-agnostic approvals could mislead HTA, potentially resulting in an overestimation of health impacts and having to deal with the uncertainties associated with heterogeneous patient outcomes. As a result, economic estimates from tissue-agnostic indications are commonly highly variable and strictly dependent on the methodology approach utilized, in the absence of comparative data [87,88].

## 5. Challenges Arising from a Lack of Data 

The challenges faced by HTA when evaluating cancer medicines and other complex health interventions have been the subject of a systematic European study undertaken by members of an umbrella organization called EUnetHTA. In this study, most EUnetHTA member organizations rated the evaluation of gene therapies and histology-agnostic indications of cancer medicines as highly challenging [89]. They noted various challenges pertaining to the assessment of the relative effectiveness and cost-effectiveness [73], including a lack of data, data immaturity, unreliable trial results, poor ad hoc post-marketing studies and non-transparent data sharing. EUnetHTA member organizations agreed that there is a need to improve the quantity and quality of data to support HTA. 

Another contributor to the lack of timely and adequate data to inform HTA is delays in or absence of post-marketing research. For example, an analysis of data sharing from pharmaceutical companies to US FDA showed a common failure in attaining the agreed deadlines for reporting the numbers and outcomes of the data requests [90]. In addition, an analysis of novel oncology medicines approved by the US FDA and EMA between 2005 and 2010 [91] displayed post-marketing studies based on efficacy endpoints only in 6.6% of the cases and 35.9% on safety. In Europe, 85% of all the medicines approved based on surrogate endpoints had not provided further trial data results on OS or QoL after a median observation period of 5.4 years post-registration [47].

## 6. What Could Be Done 

**Figure 3:**
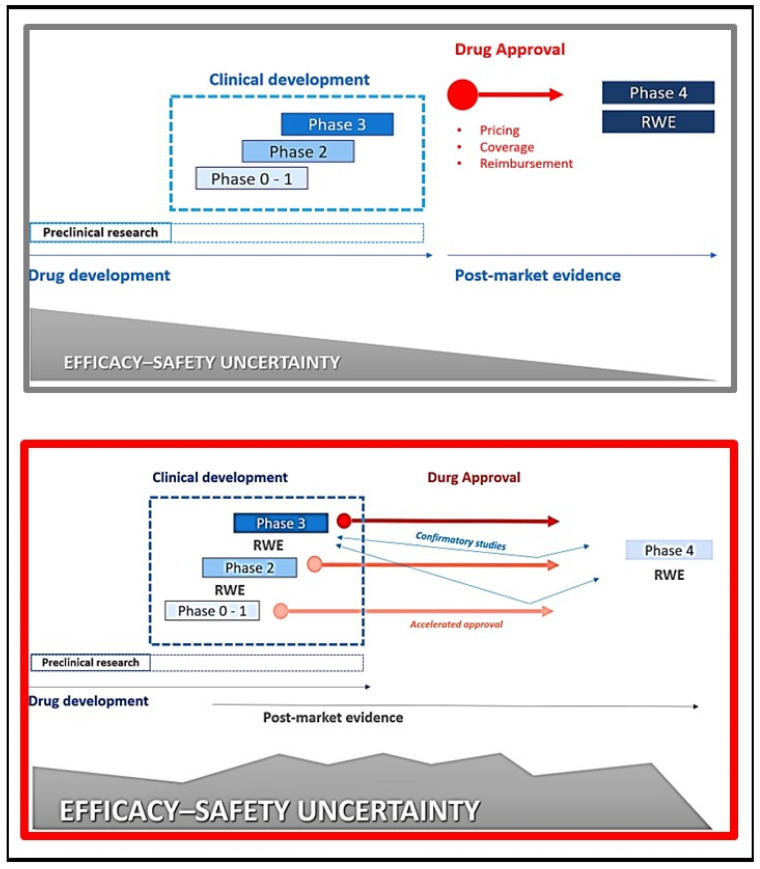
Drug development scenarios and potential impact on efficacy-safety data. In the upper panel: standard drug development; in the lower panel: potential impact of accelerated drug approvals and new trial designs on the efficacy-safety data. RWE, real-world evidence.

The absence of regulatory guidance on resolving the uncertainty that reliance on surrogate measures brings, together with the indiscriminate enthusiasm in the uptake of innovative treatments, necessitates reforming pharmaceutical policy. Uncertainty is inherent in expedited pre-approval drug testing, for too much uncertainty harms patients or wastes resources that could have been invested elsewhere [16], as shown in Figure 3.

### 6.1. Policy Harmonization between Regulatory and HTA Bodies

The development of harmonized requirements for regulatory approvals and HTA can serve to provide clarity and coordination in decision-making. One such approach is presented in the Pharmaceutical Strategy for Europe recently launched by the European Commission [92]. The strategy is set in a multidisciplinary stakeholder framework and includes a commitment from EMA to improve its role in informing clinical trial designs via scientific advice. A major aim of the strategy is to enable parallel scientific advice on clinical study design for medicines by HTA bodies and the EMA. This would enhance the harmonization of evidence-based methodology across European countries and government authorities, including translating surrogate endpoints to patient-centric metrics [93,94]. Such an approach can improve consistency in the methodologies, especially when dealing with HTA in the context of limited efficacy-safety data. The strategy is aligned with the evidence-based European Cancer Control Plan, launched in 2021, to deliver quality, equitable, and high-impact cancer care to all [21,95].

It is worth noting that critics of the harmonization processes had expressed their concern that overarching and dominant, general decisions may be too inflexible to respond to local contexts. Therefore, some degree of autonomy is desirable to enable context-appropriate implementation–one of the core aims of HTA [21,24]. While harmonization would not overcome the uncertainties associated with data per se, by enhancing the capacity to develop timely and more robust HTA methodologies through pooling national efforts and expertise, harmonization could reduce the likelihood of HTA misinforming decisions when there are limited data. Such a policy recommendation aims to pursue consistency across the policies by improving the methodological approach and building on expertise across several countries and institutions. 

### 6.2. Generation of Post-Marketing Efficacy-Safety Data

Generation of post-marketing data from the companies for new medicines must be a prerequisite for all expedited approval pathways. Post-marketing studies might prompt regulatory re-assessments to inform disinvestments from low-value or even detrimental interventions when the performance of a specific medicine, for example, is lower than or in contrast to what was previously presented for regulatory or reimbursement decisions. However, post-market studies have not been optimally useful in informing post-market HTA and revision of decisions because of various problems, such as delays in organizing these studies, difficult enrolments, and absence of clinically meaningful outcomes [47,91,96]. The need for better elucidation of the aims and purposes of post-marketing studies and clear guidance on best practices in conducting these studies could improve the timely reassessments of the interventions [97]. Post-marketing studies should consider different health system contexts, capacity, resources, and vulnerable ethnically defined groups or minorities. In addition, regulators should ensure timely and consistent submission of updated reports from clinical trials. Regulators should also mandate follow-up studies by terminating conditional approval and limiting market access when critical requests for post-marketing evaluations have not been justifiably fulfilled. In the context or rare molecular alterations and/or rare tumors, data can be generated also in the context of regulated, high-quality registries, including for the off-label use of medicines and drug-repurposing for molecular-tumor board approaches, to enhance data generation and disengage in areas of limited clinical value [98,99,100,101]. As a result, HTA should prompt new reassessments based on the updated information, inform dis-investments from low-value interventions, or even re-assess the value of specific medicines, including by changing the coverage and reimbursement recommendations. Overall, the lack of strong policies for the post-marketing evidence generation is a determinant of limited HTA potentials of dynamic medicines reassessments in a way that could timely deliver value-driven healthcare. 

### 6.3. Managing Uncertainties through Risk-Sharing

The uncertainties in HTA due to poor data could be addressed with a risk-based approach. Risk-sharing aims to distribute the consequences of the uncertainties across multiple actors. Specifically, adequate penalties should be imposed on companies when post-market evidence demonstrates benefits lower than previously claimed or when companies do not fulfill a commitment to report post-marketing evidence generation. This could be included as part of the agreement when granting earlier access to the market based on results from earlier clinical trials or less robust clinical trial data [102].

An agreement with the payments to be subject to achieving certain pre-agreed health outcomes or other milestones is called an outcome-based agreement [19]. This means that the manufacturers and the payers agree to adjust the payment levels based on the actual health outcomes achieved. It is worth noting that such an approach requires intensive HTA capacity [103,104].

HTA can inform and orient risk-sharing decisions using risk charts and similar tools. For example, a proposed HTA approach to tackle limited evidence regarding safety-efficacy is based on probabilistic models to measure the overall risk from uncertainties [105]. Such an approach intersects HTA and value-based care in the context of limited efficacy-safety data to orient decision-makers toward the best choices. In settings of resource constraints, risk estimates of HTA could be a mechanism to ensure priority-setting towards higher-value health interventions. Lack of HTA capacity, overall, can reduce the potential to pursue value-based decisions and dis-invest from low-value care through investments in the most effective and cost-effective priority cancer interventions [9,106,107,108,109].

Implementing outcome-based systems based on HTA outputs will require reliable, transparent and solid information system infrastructures and should be expanded only in priorities for high-value interventions.

### 6.4. Value Frameworks 

More recently, the introduction of value frameworks has offered the opportunity to operationalize the HTA outcomes and accelerate progress toward priority-setting and performance-based agreements. The scale most widely utilized with HTA applications in cancer is the Magnitude of Clinical Benefit Scale (MCBS) of the European Society for Medical Oncology (ESMO) [110,111]. Validation of MCBS as a tool to harmonize regulatory decisions as well as enabling HTA decisions has been provided across multiple countries, including the UK [112], Israel [113], Austria [114], France [115,116], Switzerland [117] and Kazakhstan [9]. MCBS provides explicit scores based on a transparent methodology, helping to discern high- versus low-value medicines. MCBS could inform the degree of risk as a specific intervention is being implemented and shape the terms and conditions in risk-sharing agreements. Incorporating MCBS and other public health policy tools into HTA can accelerate the delivery of value-based healthcare and drive more efficient health expenditure with better utilization of constrained resources, as reported recently in Kazakhstan by ESMO and WHO [9,118]. Other frameworks have been developed, including the ASCO Value Framework and the Canadian Drug Assessment Framework, that explicitly incorporate oncology drug costs into value assessments, which can help prioritize oncology drugs for funding and facilitate the delivery of affordable cancer care in cost-constrained health systems [111,119,120]. In general, policy value tools can help prioritize health care investments and support HTA, portending high value for money, a pillar for public health sustainability.

HTA methods are not perfect and need to be better developed. Still, HTA will primarily benefit from better and more robust data on the safety-efficacy of health interventions. Healthcare systems will need to consider adjusting their evaluative methods and processes so that they can continue to robustly assess the value for money of new treatments and services.

#### Choosing-Wisely 

Another initiative is the Choosing Wisely campaign, which has helped raise awareness on low-value care by compiling evidence-based lists of such technologies [121,122]. This framework is used in some settings as a hard list to exclude or de-prioritize low-value interventions. The recommendations are “ready to use” and implement, potentially helping countries to dis-invest or avoid investments in low-value care, for which contemporary literature is robust enough to generalize the findings across all settings. The recommendations are pragmatic and explicit. However, changing such practices remains challenging for many local health systems [121,122,123,124]. Choosing-Wisely recommendations have been identified as priority inputs to HTA to prompt dis-investments in low-value areas, including cancer medicines [121,122].

## 7. Conclusions

The central problem with using HTA for cancer medicines to drive resource allocation decisions is the lack of robust data arising from the extensive use of surrogate endpoints in regulatory approvals, a lack of generalizability of trials to the context of use, SATs and basket clinical trials. Uncertainties in the estimates of the added benefit of health interventions affect how scarce resources should be prioritized, diluting efforts toward decision-making informed by a systematic assessment of benefits and risks and achieving equitable access to beneficial medicines. Where uncertainties exist, risk-sharing policies might help improve efficiency in resource allocation. Value-based frameworks and stakeholder networks can improve the operationalization of HTA, especially in resource-constrained settings. Improving robustness and quality of data, including through better designs of clinical trials based on patient-centered direct endpoints and post-marketing studies, will improve resource allocations and catalyze disinvestments from low-value care. There is a need to end narratives about the estimated benefits of cancer medicines entirely based on speculations and assumptions on surrogate endpoints. There is a need to shift entirely towards data-driven decision-making based on direct-OS and QoL–endpoints while encouraging research on new metrics that can accelerate progress while still measuring patient-centric endpoints [81]. The central problem of uncertainty in conducting reliable HTA will not be resolved without proactive actions. There should be reforms toward patient-centered and community-devoted health care to ultimately deliver equitable cancer care.

## Figures and Tables

**Table 1:** Key elements of the Health Technology (HTA) and of regulatory assessment.

	Regulatory Assessment	HTA Assessment
Who conducts or appraises assessment?	National and supra-national authorities	National, supra-national and sub-national authorities, local health services, academia and independent authorities
Scope of assessment	Quality Safety-efficacy	Safety-efficacy Cost and cost-effectiveness Budgetary impact Other impacts: social, ethical, legal, organizational
Technical focus of assessment	Comparative assessment is not mandatory Prioritize internal validity Endpoints: laboratory or clinical endpoints	Comparative assessment commonly mandated Prioritize external validity Endpoints: clinical endpoints (overall survival, quality of life) and economic endpoints (incremental cost-effectiveness ratio)
Assessment outcome and status	Market authorization Legally binding	Funding and reimbursement recommendations Legally binding in some countries Non-legally binding advice to decision-makers Sharing information on HTA findings

Note: The endpoints in the table are examples of the most common metrics utilized for HTA and regulatory decision-making. However, the spectrum of metrics used can be commonly broader.
